# Nuclear translocation promotes proteasomal degradation of human Rad17 protein through the N-terminal destruction boxes

**DOI:** 10.1016/j.jbc.2021.100831

**Published:** 2021-06-24

**Authors:** Yasunori Fukumoto, Masayoshi Ikeuchi, Liang Qu, Tyuji Hoshino, Naoto Yamaguchi, Yuji Nakayama, Yasumitsu Ogra

**Affiliations:** 1 Graduate School of Pharmaceutical Sciences, Chiba University, Chiba, Japan; 2 Department of Biochemistry & Molecular Biology, Kyoto Pharmaceutical University, Kyoto, Japan

**Keywords:** DNA damage response, protein degradation, checkpoint control, nuclear translocation, ATPase associated with diverse cellular activities (AAA), ATR, Rad17, 9-1-1 complex, destruction box, anaphase-promoting complex, 3SA, S348A/S351A/S356A, 3SD, S348D/S351D/S356D, APC, anaphase-promoting complex, At, *Arabidopsis thaliana*, Dr, *Danio rerio*, Gg, *Gallus gallus*, Hs, *Homo sapiens*, K/R359–363A, K359A/R360A/R361A/K362A/K363A, Mm, *Mus musculus*, ND, not determined, NPT2, neomycin phosphotransferase 2, PI, propidium iodide, UV, ultraviolet, Xl, *Xenopus laevis*

## Abstract

The ATR pathway is one of the major DNA damage checkpoints, and Rad17 is a DNA-binding protein that is phosphorylated upon DNA damage by ATR kinase. Rad17 recruits the 9-1-1 complex that mediates the checkpoint activation, and proteasomal degradation of Rad17 is important for recovery from the ATR pathway. Here, we identified several Rad17 mutants deficient in nuclear localization and resistant to proteasomal degradation. The nuclear localization signal was identified in the central basic domain of Rad17. Rad17 Δ230–270 and R240A/L243A mutants that were previously postulated to lack the destruction box, a sequence that is recognized by the ubiquitin ligase/anaphase-promoting complex that mediates degradation of Rad17, also showed cytoplasmic localization. Our data indicate that the nuclear translocation of Rad17 is functionally linked to the proteasomal degradation. The ATP-binding activity of Rad17, but not hydrolysis, is essential for the nuclear translocation, and the ATPase domain orchestrates the nuclear translocation, the proteasomal degradation, as well as the interaction with the 9-1-1 complex. The Rad17 mutant that lacked a nuclear localization signal was proficient in the interaction with the 9-1-1 complex, suggesting cytosolic association of Rad17 and the 9-1-1 complex. Finally, we identified two tandem canonical and noncanonical destruction boxes in the N-terminus of Rad17 as the *bona fide* destruction box, supporting the role of anaphase-promoting complex in the degradation of Rad17. We propose a model in which Rad17 is activated in the cytoplasm for translocation into the nucleus and continuously degraded in the nucleus even in the absence of exogenous DNA damage.

The ATR pathway is one of the major DNA damage checkpoints that safeguard genetic information within the nucleus, and Rad17 and the Rad9–Hus1–Rad1 complex (9-1-1 complex) play central roles in the activation and maintenance of the ATR pathway (1, 2). Rad17 composes a Rad17–RFC2–5 (replication factor C subunits 2–5) complex in which Rad17 replaces the RFC1 subunit of a canonical RFC complex (3). The Rad17–RFC2–5 complex interacts with the 9-1-1 complex (4, 5), and the interaction is essential for activation and maintenance of the ATR pathway. The sequence similarity with RFC subunits suggests that the C-terminus of Rad17 encodes multi α-helices that interact with other RFC subunits (6). On the C-terminal end, we previously identified a conserved motif named iVERGE, which is essential for interaction with the 9-1-1 complex (7, 8, 9). The N-terminus encodes an ATPase domain of AAA+ ATPase family (10, 11) and interacts with the 9-1-1 complex (12, 13, 14) (Fig. S1). The ATP binding, but not hydrolysis, is essential for interaction with the 9-1-1 complex, and the ATP-binding activity is dispensable for the formation of the Rad17–RFC2–5 complex (4, 5).

The wild-type Rad17 protein is mainly localized in the nucleus (15, 16), mostly in the nucleoplasm (17), and also distributed in the cytoplasm (17, 18). Upon exposure to ultraviolet (UV) or ionizing radiation, endogenous Rad17 protein forms nuclear foci (16, 19). In the S phase, Rad17 protein is localized in the replication compartment (18). Rad17-S645 phosphorylation signal was observed exclusively in the nucleus (20, 21). These findings suggest the presence of the regulation of intranuclear localization in response to DNA damage. Interestingly, the Rad17 K132E mutant, which lacks ATP-binding activity, is deficient in the nuclear translocation (14). We speculate that the interaction with the 9-1-1 complex and the nuclear translocation are orchestrated by the ATPase domain. However, to our knowledge, no specific motifs or domain structures regulating the subcellular and intranuclear localization have been identified, and regulation of subcellular localization of Rad17 is largely unknown.

The anaphase-promoting complex (APC) is one of the major ubiquitin ligases involved in cell cycle progression. Ubiquitin ligase activity is triggered by binding of one of the adaptor proteins, Cdc20 or Cdh1. The APC-dependent polyubiquitination is mediated by the destruction box, one of the motifs found in substrate proteins that are recognized by Cdc20 or Cdh1. The destruction box is directly associated with Cdc20 or Cdh1 and is involved in the spatiotemporal regulation of many cellular processes by APC. The presence of the destruction box indicates that the cellular function is regulated by proteasome-dependent proteolysis (22). A previous work showed that human Rad17 interacts with Cdh1 and is degraded in a manner dependent on APC–Cdh1 to promote recovery from the DNA damage checkpoint (19). The Rad17 Δ230–270 mutant is resistant to DNA damage-induced degradation and is stabilized. Another work reported that the Rad17 R240A/L243A mutant lacks a putative degradation box and is also stabilized (23). The exogenous expression of these mutants results in prolonged activation of the ATR pathway after genotoxic stress.

Here, we show that the central basic domain between N-terminal ATPase and C-terminal α-helical domains of Rad17 protein contains the nuclear localization signal. We also show that several Rad17 mutants that are defective in the nuclear localization are resistant to proteasomal degradation. The Rad17 Δ230–270 and R240A/L243A mutants are also deficient in the nuclear localization. These data suggest a relationship between nuclear localization and proteasomal degradation of Rad17 protein. Moreover, the Rad17 Δ230–270 and R240A/L243A mutants are deficient in interaction with the 9-1-1 complex, arguing against the previous proposal that these mutants escaped from the degradation to activate the ATR pathway. By contrast, the Rad17 mutant that lacks the nuclear localization signal is proficient in the 9-1-1 interaction, suggesting the cytoplasmic interaction of Rad17 and the 9-1-1 complex. Finally, we identified putative destruction boxes in the N-terminal region of Rad17. These results propose novel regulatory mechanisms for Rad17 underlying subcellular localization and protein stability.

## Results

### Regulation of nuclear localization of Rad17 protein

**Figure 1:**
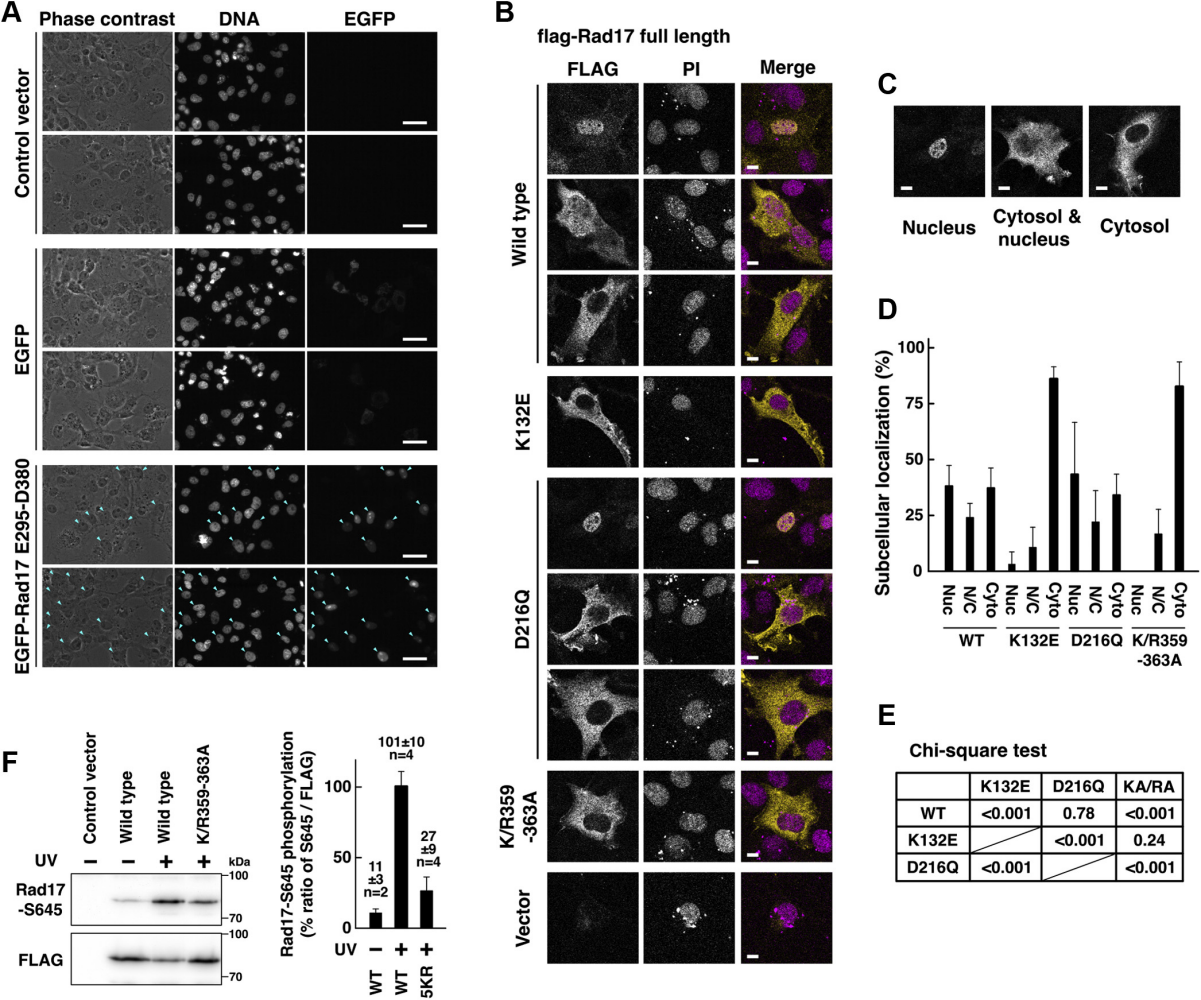
**ATP-binding activity and central basic domain of Rad17 protein are essential, but ATP hydrolysis is dispensable for nuclear localization of Rad17 protein.***A*, the central basic domain of Rad17 encoded a nuclear localization signal. Rad17 E295–D380 peptide was fused with EGFP and expressed in COS-7 cells. The cells were fixed and stained with Hoechst 33342. Representative results from two independent experiments are shown. Scale bars are 40 μm. In cells indicated by *cyan arrowheads*, EGFP-Rad17 E295–D380 was localized in the nucleus. *B*–*E*, flag-Rad17 K132E and K/R359–363A (K359A/R360A/R361A/K362A/K363A) mutants were localized in the cytoplasm. Flag-Rad17 wild-type and D216Q mutant were localized in the nucleus as well as in the cytoplasm. COS-1 cells were transfected with plasmid vectors expressing the flag-Rad17 full-length protein. After 48 h, the cells were fixed and stained with anti-FLAG antibody and propidium iodide (PI). Scale bars indicate 10 μm (*B*). Representative patterns of the localization of flag-Rad17 protein are shown. Scale bars indicate 10 μm. The “Nucleus” image is the same as that shown in panel *B* (*C*). More than 70 cells were observed for each construct, and the graph represents results from two or three independent experiments. Nuc, mostly localized in the nucleus. N/C, equally distributed in the nucleus and the cytoplasm. Cyto, mostly localized in the cytoplasm (*D*). The *p*-values of the Chi-square test of the Nuc, N/C, and Cyto classification were calculated for indicated pairs. KA/RA, K/R359–363A (*E*). *F*, the flag-Rad17 K/R359–363A mutant was deficient in Rad17-S645 phosphorylation. COS-1 cells were transfected with flag-Rad17 and irradiated with 10 J/m^2^ of UV-C. The cells were collected 3 h after irradiation and a high-salt extract was prepared. The flag-Rad17 protein was precipitated and probed with the indicated antibodies. The graph represents results from two independent experiments. n indicates the number of samples. 5KR, K/R359–363A; WT, wild type.

We examined the multiple alignment of Rad17 and RFC subunits to identify the nuclear localization signal of Rad17 and found that Rad17 has an insertion enriched with basic residues between the N-terminal ATPase and the C-terminal multi-α-helical domains, which locates in N339–D380 in human Rad17 isoform 1 (Fig. S2). This central basic domain has distributed stretches of lysine and arginine residues that are putative nuclear localization signals. A Rad17 E295–D380 peptide, which contains the central basic domain and the preceding sensor-2 helix, was fused with EGFP, and the localization was examined. The EGFP-Rad17 E295–D380 was exclusively accumulated in the nucleus (Fig. 1*A*). A stretch of five amino acids in the basic domain was replaced, and Rad17 K359A/R360A/R361A/K362A/K363A mutant, hereinafter K/R359–363A, was constructed. The flag-Rad17 K/R359–363A mutant was mostly localized in the cytoplasm (Fig. 1, *B*–*D*) and showed the same localization as the K132E mutant (Fig. 1*E*). We and others suggested that the nuclear localization is required for the Rad17-S645 phosphorylation, and the Rad17 K132E mutant is deficient in the ATR-dependent phosphorylation of Rad17-S645 (14, 20). The flag-Rad17 K/R359–363A mutant was also deficient in the Rad17-S645 phosphorylation after UV irradiation (Fig. 1*F*). The localization of Rad17 mutants was also examined in HeLa cells in which a low expression of exogenous proteins is expected because of the lack of SV40 large T antigen. The K/R359–363A mutation promoted the cytoplasmic localization of Rad17 protein (Fig. S3). These data indicate that the central basic domain of Rad17 contains the nuclear localization signal, and the K359–K363 residues encode a part of the signal.

**Figure 2:**
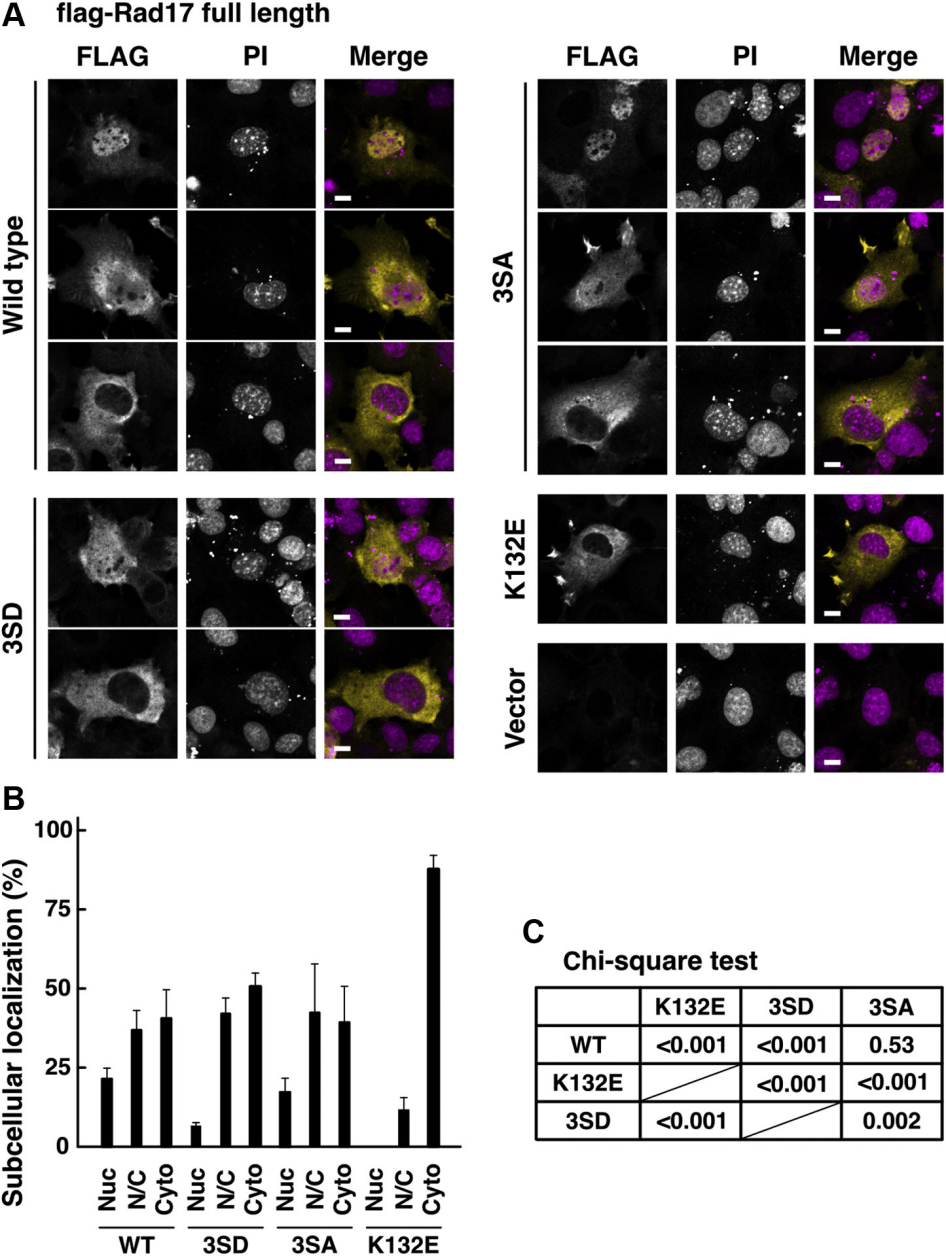
**Putative phosphorylation sites in the central basic domain of Rad17 regulate the subcellular localization and the protein amount.***A*–*C*, phosphomimetic mutations in the central basic domain of Rad17 decreased the nuclear localization. The same experiment as in Figure 1, *B*–*E*. COS-1 cells were transfected with flag-Rad17 3SD (S348D/S351D/S356D) or 3SA (S348A/S351A/S356A) mutants. The cells were fixed and stained with anti-FLAG antibody and propidium iodide (PI). Scale bars indicate 10 μm (*A*). More than 100 cells were observed for each construct, and the graph represents results from two independent experiments. Nuc, mostly localized in the nucleus. N/C, equally distributed in the nucleus and the cytoplasm. Cyto, mostly localized in the cytoplasm (*B*). The *p*-values of the Chi-square test of the Nuc, N/C, and Cyto classification were calculated for indicated pairs (*C*).

In the central basic domain of Rad17 protein, at least three phosphorylation sites were discovered and registered in the PhosphoSite plus (https://www.phosphosite.org). We examined whether these posttranslational modification sites can regulate the subcellular localization of Rad17 protein. The S348D/S351D/S356D mutation decreased the number of cells with predominant nuclear accumulation of flag-Rad17 protein (Fig. 2, *A*–*C*). The effect was statistically significant but milder than that of the K132E mutant. The S348A/S351A/S356A mutation did not affect the subcellular localization. Our data indicate that the introduction of negative charges to these residues affects the nuclear localization signal. Although the phosphorylation status of these residues under physiological conditions is currently unknown, these results raise the possibility that the potential phosphorylation sites in the central basic domain regulate the subcellular localization of Rad17 protein.

### Nuclear localization of Rad17 protein promotes its proteasomal degradation

**Figure 3:**
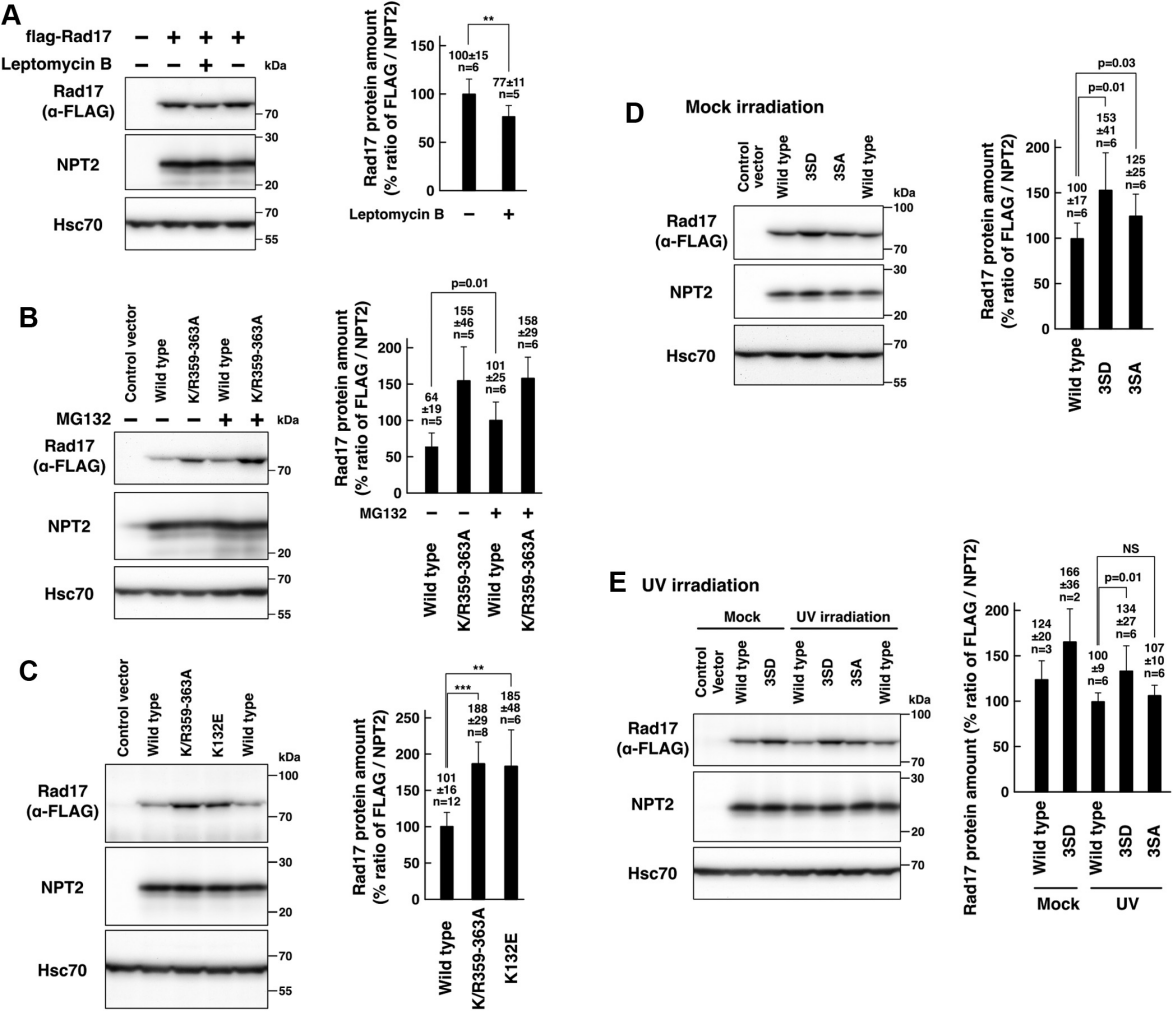
**Nuclear localization of Rad17 protein promotes its proteasomal degradation.***A*, the protein amount of Rad17 was decreased on inhibition of nuclear export. COS-1 cells were transfected with a plasmid vector expressing flag-Rad17 wild type. After 48 to 54 h, SDS-lysate was prepared and probed with the indicated antibodies. Neomycin phosphotransferase 2 (NPT2) and flag-Rad17 were expressed from the same plasmid. The cells were exposed to 5 ng/ml leptomycin B, an inhibitor of CRM1-dependent nuclear export, for 8 to 10 h before harvest, and SDS-lysate was prepared. The mock-treated sample was prepared as duplicate. The signal ratio of anti-FLAG and NPT2 blots was calculated and is shown in the graph. *B*, proteasomal degradation was involved in the increased protein amount of Rad17 K/R359–363A mutant. The same experiment as in *A* except that proteasome inhibitor MG132 was used. COS-1 cells were transfected with plasmid vectors expressing flag-Rad17 wild-type or K/R359A–363A mutant. The cells were exposed to 40 μM MG132 for 7 to 10 h before harvest, and SDS-lysate was prepared. The FLAG/NPT2 ratio of the flag-Rad17 wild-type exposed to MG132 was used as 100% standard. The Rad17 K/R359–363A mutant did not show an increase in the FLAG/NPT2 ratio upon MG132 exposure. *C*, Rad17 K/R359–363A and K132E mutants showed an increase in protein amount. The same experiment as in *A* and *B* except that inhibitors were not used. COS-1 cells were transfected with plasmid vectors expressing flag-Rad17 wild-type, K/R359A–363A mutant, or K132E mutant. The wild-type sample was prepared in duplicate. *D* and *E*, phosphomimetic mutations in the central basic domain increased Rad17 protein amount. The same experiment as in *C*. COS-1 cells were transfected with flag-Rad17 3SD or 3SA mutant, and SDS-lysate was prepared. The cells were mock-irradiated (*D*) or irradiated with 10 J/m^2^ ultraviolet (UV) and recovered for 8 to 9 h before harvest (*E*). The UV-irradiated flag-Rad17 wild-type was used as 100% standard. The graphs represent the results from more than two or three independent experiments. The *p*-values were calculated using Student’s or Welch’s *t* test. n indicates the number of samples. ∗∗∗*p* < 0.001. ∗∗*p* < 0.01. NS, not significant.

Interestingly, the cytoplasmic flag-Rad17 K/R359–363A mutant showed increased protein amount (Fig. 1*F*). The exposure to leptomycin B, an inhibitor of CRM1-dependent nuclear export, resulted in a slight but significant decrease of flag-Rad17 protein amount and FLAG/NPT2 ratio (Fig. 3*A*). Flag-Rad17 and neomycin phosphotransferase 2 (NPT2) were simultaneously expressed from the same plasmid vector, and the expression level of flag-Rad17 was examined in the panel of NPT2. The flag-Rad17 K/R359–363A mutant showed an increase in the FLAG/NPT2 ratio (Fig. 3*B*). In addition, on exposure to proteasome inhibitor MG132, flag-Rad17 wild-type protein showed an increase in the FLAG/NPT2 ratio; however, the flag-Rad17 K/R359–363A mutant showed no increase in the FLAG/NPT2 ratio (Fig. 3*B*). The flag-Rad17 wild-type and the K/R359–363A mutant showed a half-life of 7.9 and 34.7 h, respectively (Fig. S4). These indicate that the increased protein amount of flag-Rad17 K/R359–363A mutant is due to the inhibition of its proteasomal degradation, suggesting that the nuclear localization of flag-Rad17 protein promotes its proteasomal degradation and that cytoplasmic Rad17 is not degraded by the proteasome. This is supported by the findings that the S348D/S351D/S356D mutation, which causes cytoplasmic localization (Fig. 2), resulted in an increase in the flag-Rad17 protein amount and the FLAG/NPT2 ratio (Fig. 3*D*). Upon UV irradiation, the S348D/S351D/S356D mutant but not the S348A/S351A/S356A mutant showed an increase in the protein amount and the FLAG/NPT2 ratio, although the extent of increase was lesser than that for the mock-irradiated sample (Fig. 3*E*). These findings also exclude the possibility that the K/R359–363A mutation may replace lysine residues that serve as ubiquitination sites for proteasomal degradation.

**Figure 4:**
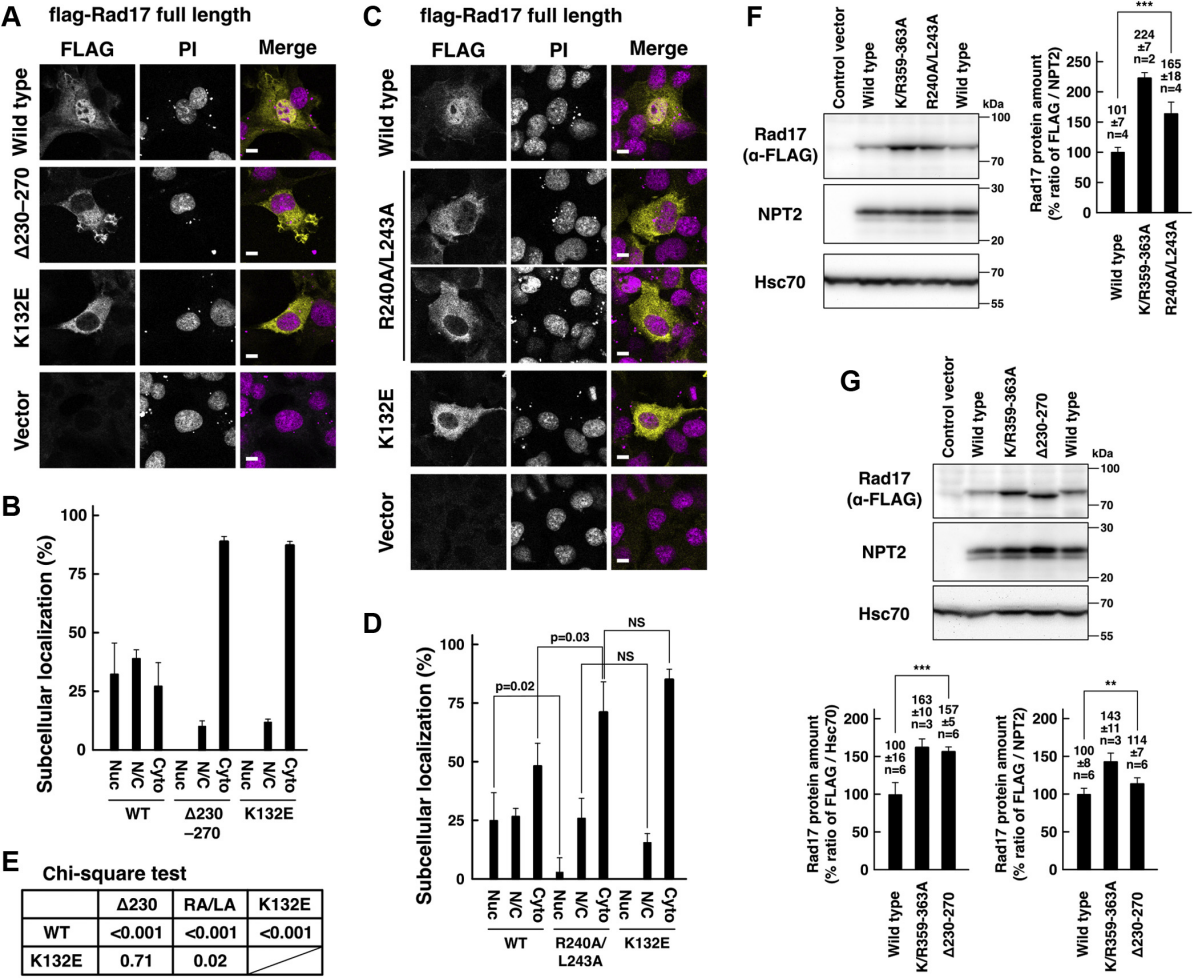
**Stabilization of Rad17Δ230–270and R240A/L243A mutants is due to deficient nuclear translocation.***A*–*E*, Rad17 Δ230–270 and R240A/L243A mutants were deficient in nuclear localization. The same experiment as in Figure 1, *B*–*E*. COS-1 cells were transfected with flag-Rad17 Δ230–270 or R240A/L243A mutant. The cells were fixed and stained with anti-FLAG antibody and propidium iodide (PI). Scale bars indicate 10 μm (*A* and *C*). The graphs represent results from two (*B*) or three (*D*) independent experiments. More than 100 cells were observed for each construct. The *p*-values indicated in the graphs were calculated using Student’s *t* test. Nuc, mostly localized in the nucleus. N/C, equally distributed in the nucleus and the cytoplasm. Cyto, mostly localized in the cytoplasm (*B* and *D*). The *p*-values of the Chi-square test of the Nuc, N/C, and Cyto classification were calculated for indicated pairs. RA/LA, R240A/L243A (*E*). The flag-Rad17 Δ230–270 mutant showed the same subcellular localization as the K132E mutant (*A*, *B* and *E*). The R240A/L243A mutant showed a decrease in the nuclear localization and an increase in the cytoplasmic localization (*C* and *D*). *F* and *G*, Rad17 Δ230–270 and R240A/L243A mutants showed increased protein amounts as reported previously. The same experiment as in Figure 3. COS-1 cells were transfected with flag-Rad17 Δ230–270 or R240A/L243A mutants, and SDS-lysate was prepared. The signal ratios of anti-FLAG and NPT2 blots are shown in graphs. The graphs represent results from two (*F*) or three (*G*) independent experiments. FLAG/Hsc70 ratio is also shown (*G*). The *p*-values were calculated using Student’s or Welch’s *t* test. n indicates the number of samples. ∗∗∗*p* < 0.001. ∗∗*p* < 0.01. NS, not significant.

We found previously that the flag-Rad17 K132E mutant showed an increase in the protein amount (14). The flag-Rad17 K132E mutant showed an increase in the protein amount and the FLAG/NPT2 ratio to the same extent as the K/R359–363A mutant (Fig. 3*C*). In previous works, the Rad17 Δ230–270 and R240A/L243A mutants were reported to show resistance to proteasomal degradation and increased protein stability after UV irradiation (19, 23). We examined the subcellular localization of the Rad17 Δ230–270 and R240A/L243A mutants. As expected, the flag-Rad17 Δ230–270 mutant was exclusively localized in the cytoplasm and showed the same result as the K132E mutant (Fig. 4, *A*, *B* and *E*). The R240A/L243A mutant showed a decrease in the number of cells with nuclear localization of the flag-Rad17 (Nuc) and an increase in the cytosolic localization (Cyto), although it still showed the same percentage of cells with the nuclear/cytosolic localization (N/C) as the wild type (Fig. 4, *C* and *D*). The flag-Rad17 R240A/L243A and Δ230–270 mutants showed an increase in the protein amount and the FLAG/NPT2 ratio as reported previously (Fig. 4, *F* and *G*). These results clearly demonstrate that Rad17 protein level is regulated by its subcellular localization in a proteasome-dependent manner.

The increase of the FLAG/NPT2 ratio of the Rad17 Δ230–270 mutant was smaller than that of other mutants (Fig. 4*G*). This would be because the amount of NPT2 was also increased upon the expression of Rad17 Δ230–270. The previous work did not use transfection controls such as NPT2 (19). Another difference is that the cells were UV-irradiated and mock-irradiated in the previous and current studies, respectively.

### ATP binding, but not ATP hydrolysis, is essential for nuclear localization of Rad17 protein

We previously reported that the Rad17 K132E mutant, which lacks ATP-binding activity, is deficient in the nuclear translocation and localizes in the cytoplasm (14). We, thus, examined whether the ATP binding or hydrolysis is required for the nuclear localization. We confirmed that the flag-Rad17 K132E mutant, which is deficient in ATP binding, was localized in the cytoplasm (Fig. 1, *B*–*E*). The flag-Rad17 D216Q mutant, which is proficient in ATP binding but deficient in hydrolysis, localized in both the nucleus and the cytoplasm (Fig. 1, *A*–*C*) and showed the same localization as the wild type (Fig. 1*D*). These indicate that the ATP-binding activity, but not the hydrolysis activity, is essential for nuclear localization of Rad17 protein.

**Figure 5:**
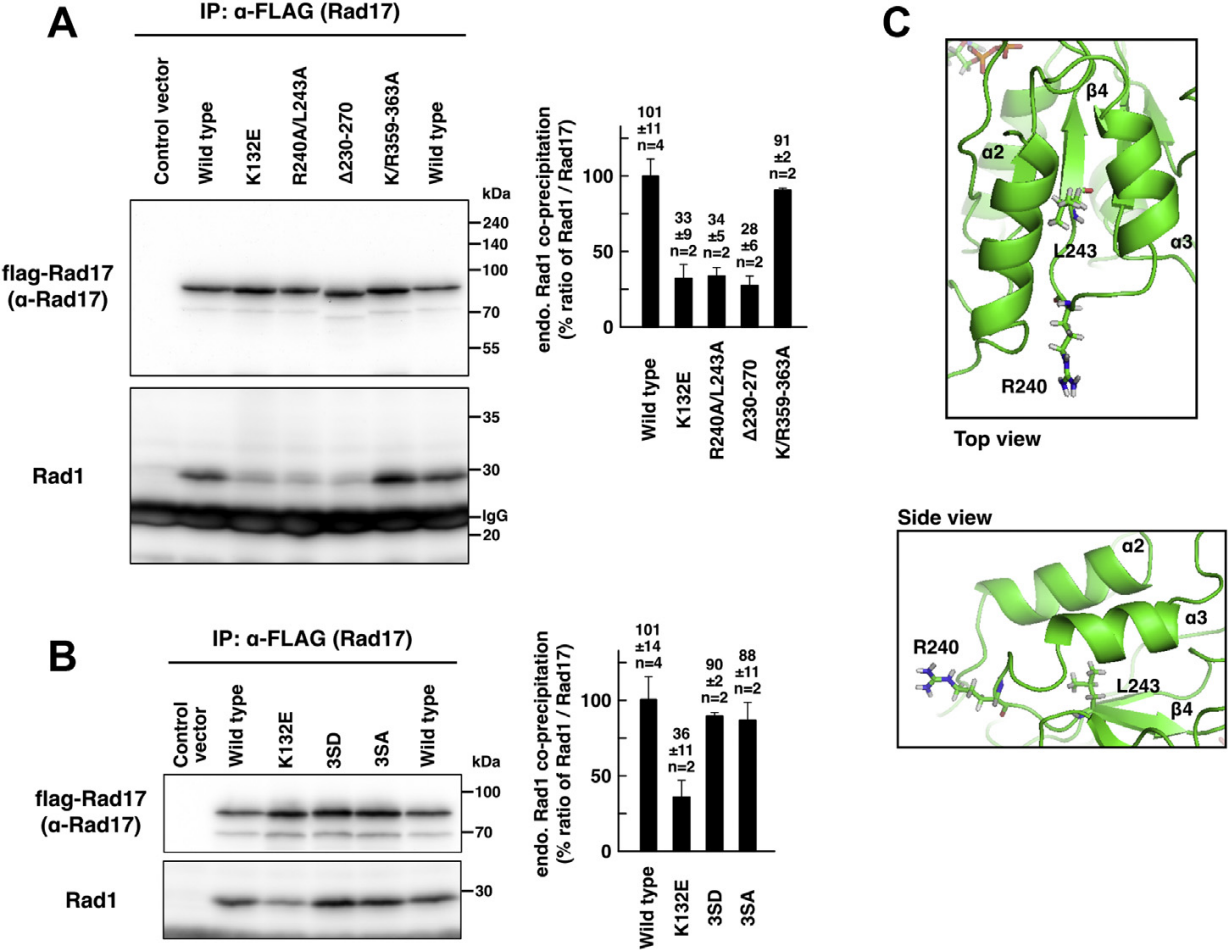
**Rad17Δ230–270and R240A/L243A mutants have a structural abnormality in the ATPase domain.***A* and *B*, Rad17 Δ230–270 and R240A/L243A mutants were deficient, and K/R359–363A and 3SD mutants were proficient in the interaction with the 9-1-1 complex. flag-Rad17 was expressed in COS-1 cells, and a low-salt extract was prepared. flag-Rad17 was precipitated, and the coprecipitation of endogenous Rad1 was examined. The signal intensity of endogenous Rad1 was normalized to that of flag-Rad17 and is shown in graphs. The graphs represent results from two independent experiments. n indicates the number of samples. Rad17 Δ230–270 and R240A/L243A mutants did not coprecipitate endogenous Rad1. Rad17 K132E was used as negative control. *C*, structural modeling of ATPase domain of human Rad17. Structure of the ATPase domain of Rad17 was modeled based on the homology with RFC1. The drawing represents a snapshot structure of the R240 and L243 residues at the last of the 100 ns MD simulation. The numbering of α-helices and β-strands was assigned according to reference (25).

The Rad17 Δ230–270 mutant, which was exclusively localized in the cytoplasm (Fig. 4*A*), lacks the sensor 1 sequence of the AAA+ ATPase domain. This raises the possibility that the Δ230–270 mutant has a structural abnormality in the ATPase domain. The ATP-binding activity of Rad17 is essential for interaction with the 9-1-1 complex (4, 5). In this regard, we examined the interaction between Rad17 and the 9-1-1 complex to check the structural integrity. We used a low-salt extract depleted of chromatin-binding proteins. The flag-Rad17 K132E mutant failed to precipitate endogenous Rad1, as reported previously (Fig. 5*A*). The flag-Rad17 Δ230–270 and R240A/L243A mutants were also unable to precipitate Rad1, indicating that these mutants were deficient in the interaction with the 9-1-1 complex. In the ATPase domain (N77–N338), the 230 to 270 residues encompass the two α-helices and one β-strand (Fig. S5), and the Δ230–270 mutation deleted the canonical feature of the AAA+ ATPases. We speculate that these mutants have a structural abnormality in the ATPase domain. Contrary to our expectations, the cytoplasmic flag-Rad17 K/R359–363A mutant normally precipitated Rad1 (Fig. 5*A*). The S348D/S351D/S356D and S348A/S351A/S356A mutants also normally precipitated Rad1 (Fig. 5*B*). These data indicate that the nuclear translocation is dispensable for interaction with the 9-1-1 complex.

### Rad17 has two putative destruction boxes

A previous work reported that the Rad17 R240A/L243A mutant lacks a putative degradation box (23). We modeled a structure of human Rad17 based on the homology with RFC1, whose structure was reported as a canonical RFC1–5 complex (6). Rad17 R240 was placed on a loop between α3 helix and β4 strand, and then R240 was positioned on the protein surface and exposed to solvent (Fig. 5*C*). The L243 residue was placed on β4 strand and surrounded by β4 strand and α2 and α3 helices. The L243 residue occupied the same position during the 100 ns MD simulation (data not shown). These results indicate that L243 is folded in the interior of Rad17 protein and that the local structure is stable. It suggests that the R240–L243 sequence is less likely to be recognized by Cdh1, although it matches the RxxL consensus of the destruction box.

**Figure 6:**
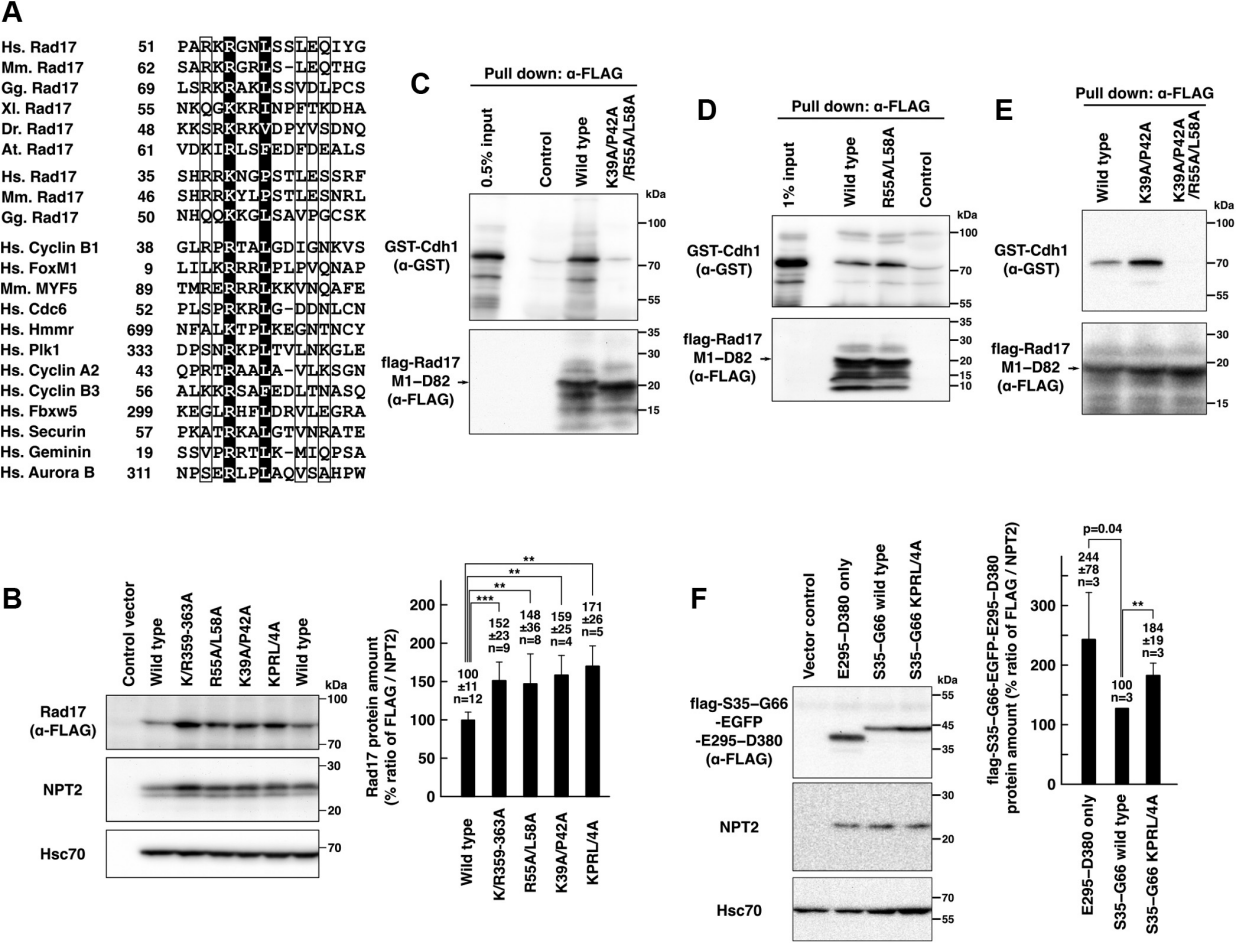
**Human Rad17 R53–Q63 and R37–S47 residues encode the destruction box.***A*, amino acid sequences in Rad17 R53–Q63 and R37–S47 showed similarity to the canonical destruction box. The putative destruction boxes from Rad17 proteins were aligned with the destruction boxes known in mammalian proteins. *White letters* on *black background* indicate the consensus residues, which are R55/L58 and K39/P42 in human Rad17 (isoform 1). *Letters* in frames also indicate the conserved residues. *B*, Rad17 R55A/L58A and K39A/P42A mutants showed increases in protein amount. The same experiments as in Figure 3. COS-1 cells were transfected with flag-Rad17 R55A/L58A, K39A/P42A, and K39A/P42A/R55A/L58A (KPRL/4A) mutants, and SDS-lysate was prepared. The signal ratio of anti-FLAG and NPT2 blots is shown in a graph. The graph represents the result from three independent experiments. The *p*-values were calculated using Welch’s *t* test. *C*–*E*, Rad17 has two putative destruction boxes on the N-terminus. Recombinant proteins of GST-Cdh1 and flag-Rad17 M1–D82 peptides were separately expressed in bacteria. The lysates were mixed and incubated. The flag-Rad17 M1–D82 peptide was precipitated, and coprecipitation of GST-Cdh1 was examined with anti-GST blot. Mutation in K39A/P42A/R55A/L58A (*C*), but not R55A/L58A (*D*) or K39A/P42A (*E*), abolished the interaction. The representative results from more than two independent experiments are shown. *F*, the Rad17 S35–G66 peptide was fused with flag tag, EGFP, and the E295–D380 peptide and was expressed in COS-1 cells (flag-S35–G66-EGFP-E295–D380). Forty-eight hours after transfection, the SDS lysate was prepared and probed with the indicated antibodies. The graph shows the signal ratios of anti-FLAG and NPT2 blots in three independent experiments. The *p*-values were calculated using the paired *t* test. The S35–G66 wild-type was used as 100% standard. E295–D380 only, flag-EGFP-E295–D380. S35–G66 wild-type, flag-S35–G66-EGFP-E295–D380 with the wild-type destruction boxes. S35–G66 KPRL/4A, flag-S35–G66-EGFP-E295–D380 with the mutated destruction boxes. n indicates the number of samples. ∗∗*p* < 0.01. ∗∗∗*p* < 0.001. At, *Arabidopsis thaliana*; Dr, *Danio rerio*; Gg, *Gallus gallus*; Hs, *Homo sapiens*; Mm, *Mus musculus*; Xl, *Xenopus laevis*.

In human Rad17 (isoform 1), the sequence homology suggested that R55 and L58 residues, which are on an N-terminal peptide protruding from the ATPase domain (10, 11), match the RxxL consensus of the destruction box. In addition, we noticed that Rad17 has another sequence similar to the destruction box in R37–S47 residues in which K39 and P42 occupy the RxxL consensus (Fig. 6*A*). The multiple alignment suggests that the position of L61 is preferentially occupied by hydrophobic residues in the other destruction boxes (Fig. 6*A*) (http://slim.ucd.ie/apc/index.php), which interact with a hydrophobic patch on Cdh1 (24). The positions of R53 and Q63 were respectively preferred by lysine/arginine and basic residues (Fig. 6*A*) (http://slim.ucd.ie/apc/index.php). These data raise the possibility that the Rad17 R53–Q63 and R37–S47 residues encode the destruction boxes.

We examined whether the R53–Q63 and K39–P42 residues are involved in the degradation of Rad17 protein. In the flag-Rad17 full-length protein, the R55A/L58A mutation increased the protein amount and the FLAG/NPT2 ratio to the same extent as the K/R359–363A mutation (Fig. 6*B*). The K39A/P42A and K39A/P42A/R55A/L58A mutations also increased the protein amount and the FLAG/NPT2 ratio, indicating that the R55A/L58A and K39A/P42A mutations stabilize Rad17 protein. We examined the interaction between human Cdh1 and a peptide derived from Rad17 M1–D82 that contains the destruction box. GST-Cdh1 and flag-Rad17 M1–D82 were expressed in bacteria and used for the pull-down assay. The Rad17 M1–D82 peptide precipitated GST-Cdh1. The K39A/P42A/R55A/L58A mutation abolished the coprecipitation of GST-Cdh1 (Fig. 6*C*). The R55A/L58A or K39A/P42A mutations did not inhibit the coprecipitation of GST-Cdh1 with the Rad17 M1–D82 peptide (Fig. 6, *D* and *E*). These data suggest that human Rad17 has two tandem destruction boxes on the N-terminus. We also examined whether the N-terminal destruction boxes promote the proteasomal degradation of exogenous proteins. The Rad17 S35–G66 peptide was fused with EGFP, and the stability of the recombinant protein was examined *in vivo*. The insertion of the destruction boxes decreased the FLAG/NPT2 ratio, and the K39A/P42A/R55A/L58A mutations reversed the effect (Fig. 6*F*), suggesting that the destruction boxes in the S35–G66 peptide destabilized the EGFP protein. These data demonstrated that the N-terminus of Rad17 contains destruction boxes.

**Figure 7:**
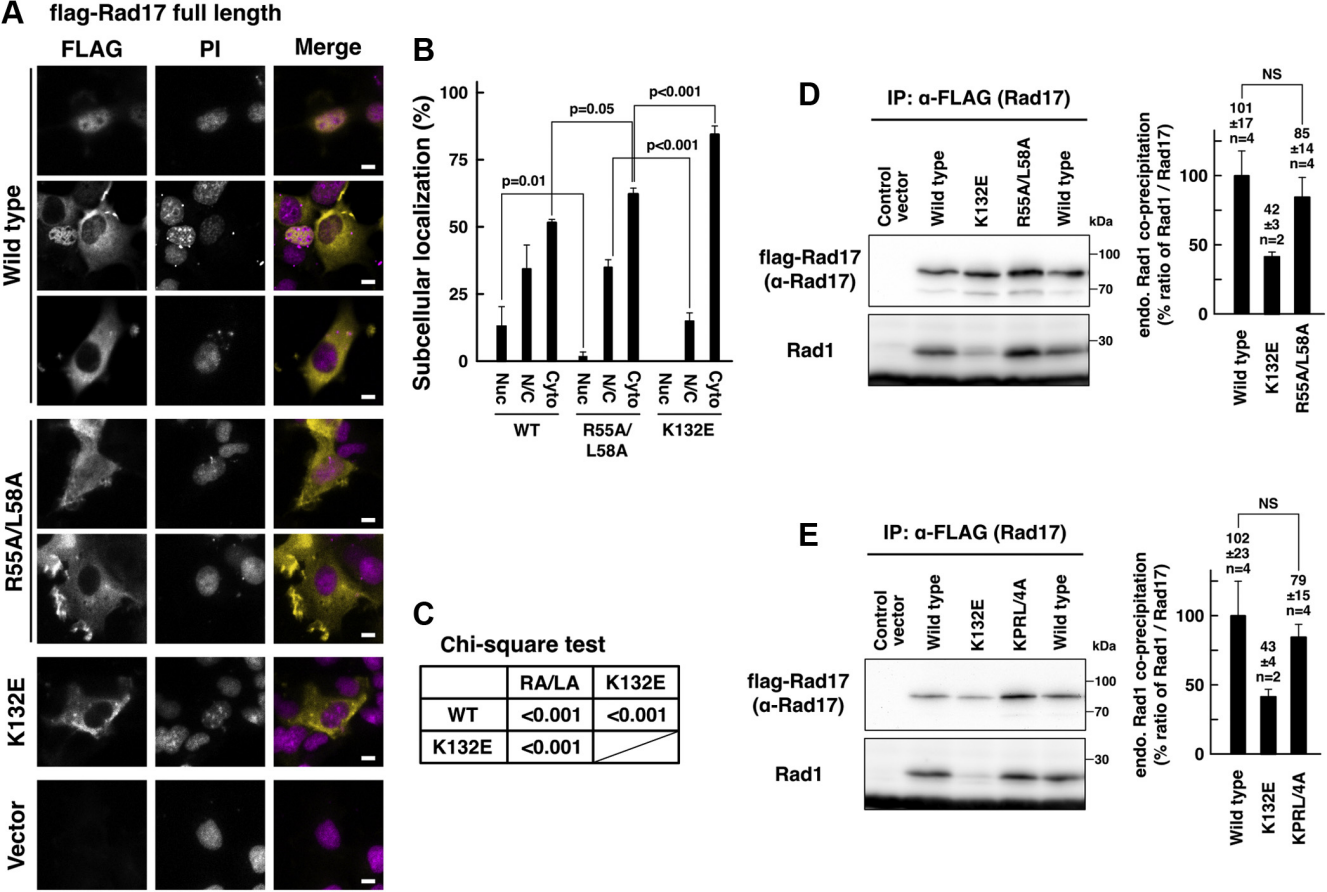
**Mutations in the destruction box do not affect the overall structure of the ATPase domain of Rad17.***A*–*C*, Rad17 R55A/L58A mutant was proficient in the nuclear translocation. The same experiment as in Figure 1, *B*–*E*. COS-1 cells were transfected with flag-Rad17 R55A/L58A mutant, and the cells were fixed and stained. Scale bars indicate 10 μm (*A*). The graph represents results from more than three independent experiments. More than 100 cells were observed for each construct. The *p*-values indicated in the graph were calculated using Student’s *t* test. Nuc, mostly localized in the nucleus. N/C, equally distributed in the nucleus and the cytoplasm. Cyto, mostly localized in the cytoplasm (*B*). The *p*-values of the Chi-square test of the Nuc, N/C, and Cyto classification were calculated for indicated pairs. RA/LA, R55A/L58A (*C*). The flag-Rad17 R55A/L58A mutant localized in the nucleus and the cytoplasm. *D* and *E*, Rad17 R55A/L58A and K39A/P42A/R55A/L58A mutants were proficient in interaction with the 9-1-1 complex. The same experiment as in Figure 5*A*. COS-1 cells were transfected with flag-Rad17 R55A/L58A or K39A/P42A/R55A/L58A (KPRL/4A) mutant, and the low-salt extract was prepared. The coprecipitation of endogenous Rad1 with flag-Rad17 was examined. The graphs represent results from more than two independent experiments. n indicates the number of samples. NS, not significant.

We examined the subcellular localization of the R55A/L58A mutant to exclude the possibility that the protein amounts of these mutants may be increased by their cytoplasmic localization. The wild-type and the R55A/L58A mutant showed the same percentage of cells with the nuclear/cytosolic localization (N/C), whereas the R55A/L58A mutant showed a decrease in the number of cells with predominant nuclear localization of flag-Rad17 protein (Nuc) (Fig. 7, *A* and *B*). The effect was milder than that of the K132E mutant (Fig. 7*C*). These indicate that the R55A/L58A mutant was translocated into the nucleus.

We also examined the interaction with the 9-1-1 complex. Unlike the Δ230–270 and R240A/L243A mutants, the R55A/L58A and K39A/P42A/R55A/L58A mutants normally precipitated Rad1 (Fig. 7, *D* and *E*). This indicates that the R55A/L58A and K39A/P42A/R55A/L58A mutations do not affect the interaction with the 9-1-1 complex. As the ATPase domain interacts with the 9-1-1 complex (12, 13, 14), these data suggest that these mutations do not affect the overall structure and ATP-binding activity of the ATPase domain of Rad17 and indicate that the increase in the protein amount of these mutants is independent of the ATPase domain. Taken together, these results suggest that the K39/P42/R55/L58 residues play a role in the Rad17 degradation as the destruction boxes.

## Discussion

The DNA damage checkpoint is regulated by various mechanisms, including subcellular localization and proteasomal degradation of the component proteins. It has not been reported whether the subcellular localization of Rad17 may play a role in the DNA damage checkpoint responses. Here we showed that the nuclear translocation promotes proteasomal degradation of Rad17 protein and that the destruction box is located on the N-terminal protruding peptide of Rad17.

### Nuclear translocation and proteasomal degradation of Rad17 protein

**Figure 8:**
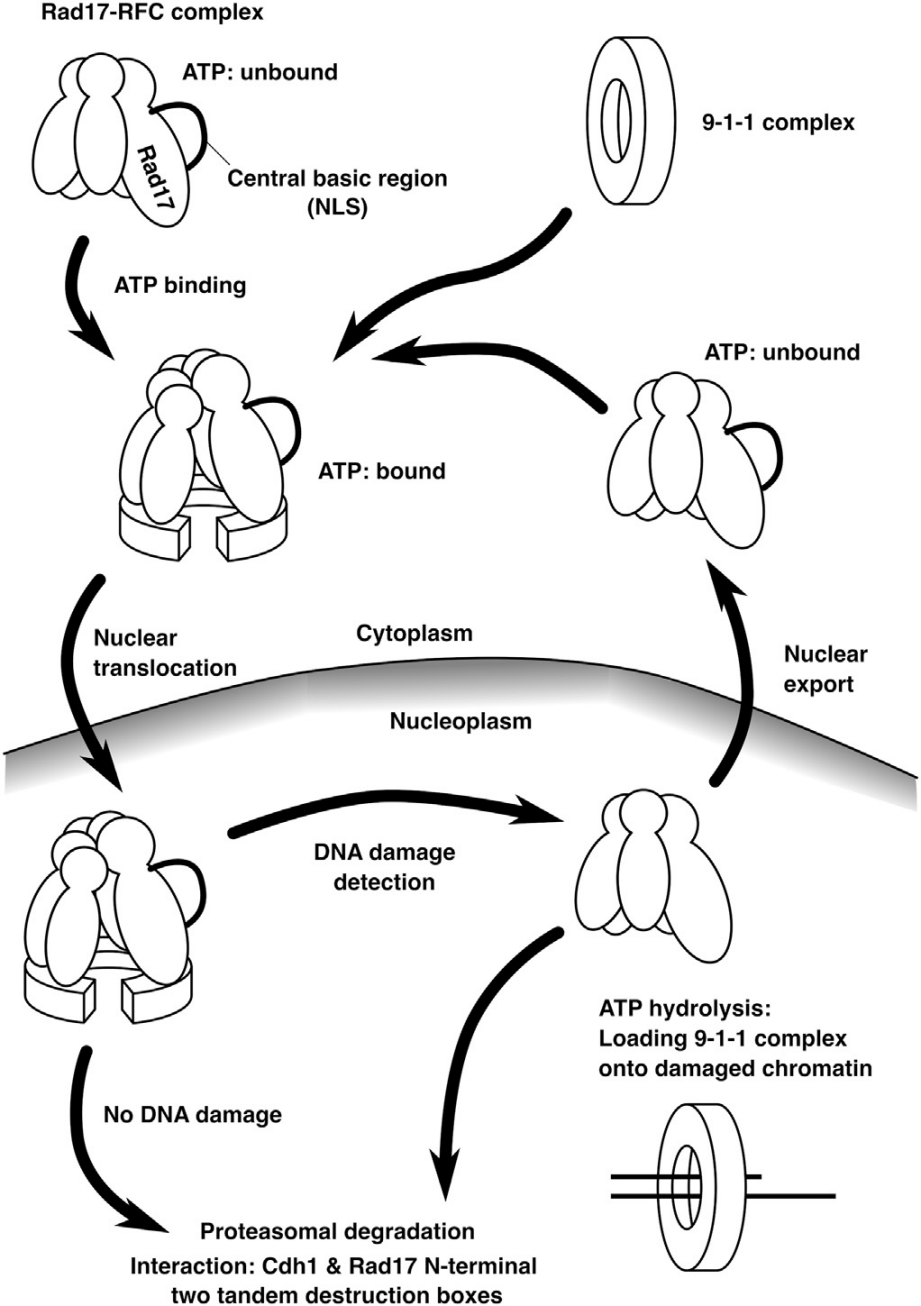
**A proposed model.** A schematic model of nuclear translocation and proteasomal degradation of Rad17. In the cytoplasm, Rad17 changes its conformation to associate with the 9-1-1 complex upon ATP binding and translocates into the nucleus. In the nucleus, Rad17 is degraded through interaction between Cdh1 and the N-terminal destruction boxes. Alternatively, Rad17 releases ATP/ADP and is exported from the nucleus.

Previous works demonstrated that APC–Cdh1 is involved in the ubiquitination and proteasomal degradation of Rad17 (19, 23). However, there was no mention of the subcellular localization of Rad17 mutants. Here, we identified several Rad17 mutants that are deficient in the nuclear accumulation (Figs. 1, 2 and 4). These mutants showed increased protein amounts (Figs. 3 and 4) as a result of escape from proteasomal degradation (Fig. 3*B*). Our results indicate that the nuclear localization of Rad17 is important for the proteasomal degradation. A previous work showed that Cdh1 has a nuclear localization signal and accumulates in the nucleus (25). One possible explanation is that the cytoplasm-localized Rad17 protein is physically taken away from Cdh1, thereby escaping from the degradation. Whereas previous works mainly focused on the Rad17 degradation induced by DNA damage induction (19, 23), we showed that Rad17 is also constitutively degraded in mock-irradiated cells in a manner dependent on the nuclear translocation (Figs. 3 and 4). Our data and those of others indicate that Rad17 is synthesized and transported into the nucleus for degradation. We propose that Rad17 protein is constitutively degraded to suppress aberrant activation of the DNA damage checkpoint. Previous work showed an increase in Rad17 protein amount after UV irradiation (19), and we speculate that the degradation is suppressed in the presence of genotoxic stress to ensure rapid response, more rapid than the transcriptional activation of *Rad17* gene, to the genotoxic stress (Fig. 8).

We previously showed that the Rad17 K132E mutant localized in the cytoplasm; however, the underlying mechanism was unclear. We showed here that the nucleotide-binding activity, but not hydrolysis, is important for the nuclear accumulation (Fig. 1, *B* and *D*). The ATP binding is also essential for interaction with the 9-1-1 complex (Fig. 5, *A* and *B*) (4, 5), suggesting a possible link between the interaction with the 9-1-1 complex and the nuclear translocation of Rad17 protein. However, we previously showed that the Rad17 ΔC14, Rad17-Y665E, and Rad17-Y195E mutants are defective in the interaction with the 9-1-1 complex but are proficient in the nuclear localization (7, 14). This suggests that the nuclear localization of Rad17 is independent of the interaction with the 9-1-1 complex. The Rad17 K/R359–363A and S348D/S351D/S356D mutants coprecipitated Rad1 (Fig. 5, *A* and *B*), indicating that the nuclear translocation is dispensable for interaction between the Rad17–RFC2–5 and 9-1-1 complexes. These point to a possible model in which the Rad17–RFC2–5 complex binds ATP to capture the 9-1-1 complex in the cytoplasm and then translocates into the nucleus (Fig. 8). By analogy with a canonical RFC complex, Rad17 hydrolyzes ATP on the chromatin, and the 9-1-1 complex is locked on the DNA. We hypothesize that Rad17 protein is inactivated in two pathways after loading of the 9-1-1 complex (Fig. 8): In one pathway, Rad17 is kept in the nucleus and ubiquitinated for proteasomal degradation. In another pathway, Rad17 is exported to the cytoplasm and reused. As endogenous Rad17 protein was observed mostly in the nucleus (15, 16), the former pathway would be dominant.

### Rad17 Δ230–270 and R240A/L243A mutants

The APC/Cdh1-dependent degradation of Rad17 was reported previously. The Rad17 Δ230–270 and R240A/L243A mutants induced prolonged Chk1-S345 phosphorylation, retardation in the subsequent mitotic entry, and delay in inactivation of the DNA damage checkpoint (19, 23). However, the Rad17 Δ230–270 and R240A/L243A mutants failed to interact with the 9-1-1 complex (Fig. 5, *A* and *B*), and the Δ230–270 mutant was also unable to translocate into the nucleus (Fig. 4, *A* and *B*). These data indicate that the persistent checkpoint activation is induced by aberrant checkpoint responses, possibly through replication fork collapse. As the cytoplasmic Rad17 K/R359–363A and S348D/S351D/S356D mutants normally interacted with the 9-1-1 complex (Fig. 5, *A* and *B*), the deficient interaction of the Rad17 Δ230–270 and R240A/L243A mutants is not due to the deficient nuclear translocation. The ATP binding induces conformational changes in Rad17 protein and is essential for interaction with the 9-1-1 complex (4, 5). The Δ230–270 mutant has a large deletion in the AAA+ ATPase domain of Rad17, which expands N77–N338 residues. The R240A/L243A mutant has a mutation in the conserved leucine in the sensor 1 sequence, which results in loss or decrease of ATPase activity in AAA+ ATPases (26). One possible explanation is that the Δ230–270 and R240A/L243A mutants, as well as the K132E mutant, are in the ATP-unbound conformation that is unable to interact with the 9-1-1 complex. As the ATP-binding activity is essential for the nuclear translocation (Fig. 1, *B* and *D*), it also explains the cytoplasmic localization of the Δ230–270 and R240A/L243A mutants.

### Destruction boxes in Rad17

**Figure 9:**
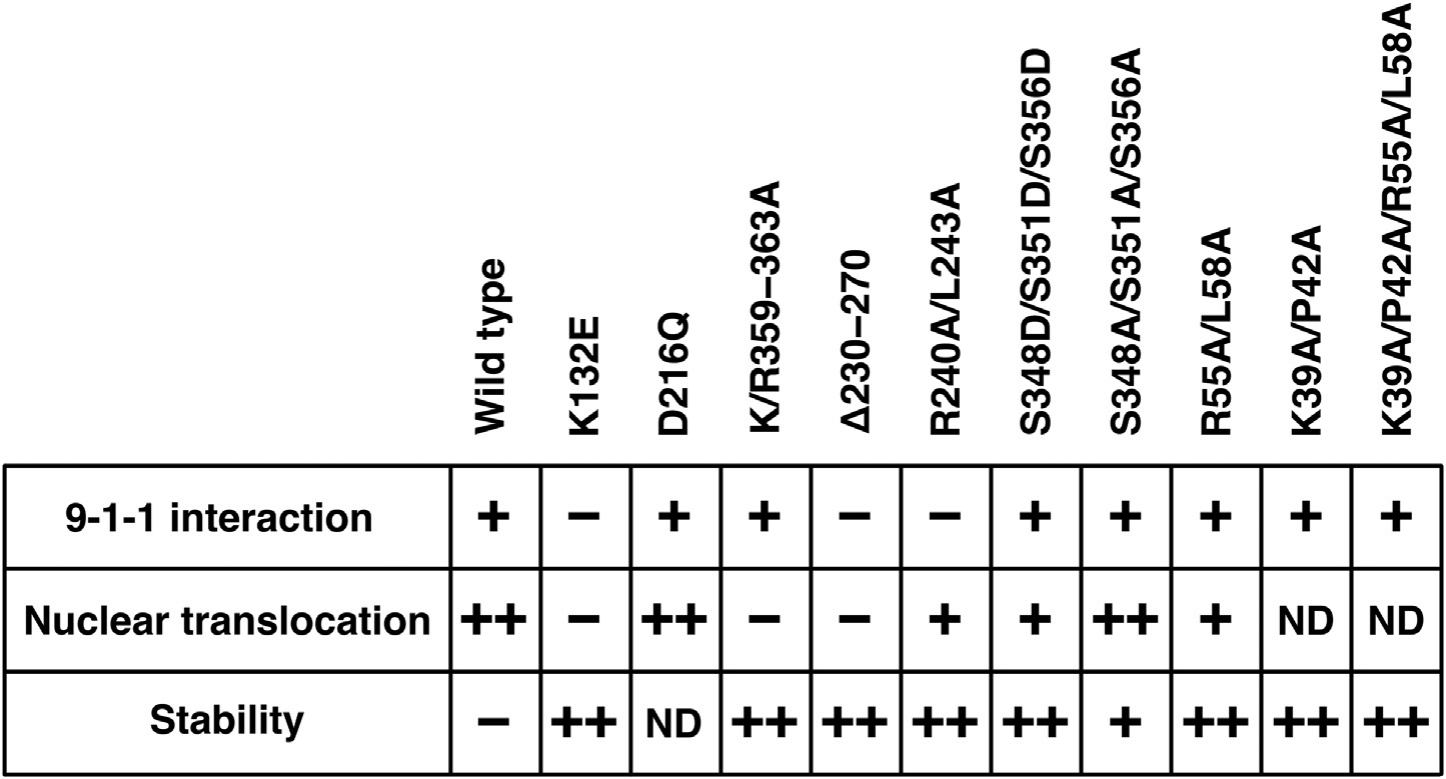
**Characteristics of Rad17 mutants are summarized.** Interaction with the 9-1-1 complex, nuclear translocation, and stability of the Rad17 protein are shown. ND, not determined.

Proteasomal degradation of Chk1 has been reported and is related to the recovery from the DNA damage checkpoint (27, 28, 29, 30). It is reasonable to speculate that the proteasomal degradation of Rad17 is important for the checkpoint recovery. However, to our knowledge, Rad17 mutants that are defective in the interaction with APC/Cdh1 have not been identified so far. The characteristics of Rad17 mutants are summarized in Figure 9. The Rad17 R55A/L58A mutant showed an increase in protein amount, translocated into the nucleus, and interacted with the 9-1-1 complex (Figs. 6 and 7). Furthermore, the Rad17 M1–D82 peptide interacted with Cdh1 *in vitro*, and the interaction was inhibited by the K39A/P42A/R55A/L58A mutation (Fig. 6, *C*–*E*). These data indicate that the Rad17 N-terminal peptide contains the *bona fide* destruction box. As the interaction was not inhibited by the R55A/L58A or K39A/P42A mutations, we propose that Rad17 has two tandem destruction boxes that have canonical and noncanonical sequences centered by R55/L58 and K39/P42 residues, respectively. Currently, most of the experimentally confirmed destruction boxes are in the canonical RxxL consensus. It will be essential to find the noncanonical destruction boxes in other proteins.

## Conclusion

We showed here that the proteasomal degradation of Rad17 is regulated by the subcellular localization and that the localization of Rad17 protein should be taken into consideration in analyzing the mechanisms of inactivation of the ATR pathway. The factors that affect the subcellular localization of Rad17 include the nucleotide-binding activity, the central basic domain, and the phosphorylation sites in the central basic domain. We also identified the destruction boxes in the Rad17 N-terminus. Our data suggest a model in which capture of the 9-1-1 complex and translocation into the nucleus are orchestrated by the ATP binding of Rad17, and Rad17 is inactivated in the nucleus by the proteasomal degradation and nuclear export. These constitutive production and degradation processes enable a rapid increase in Rad17 protein in response to genotoxic stress by inhibiting the degradation. The constitutive degradation also suppresses the basal amount of Rad17 protein, which will prevent the aberrant activation of the ATR pathway. Although preceding works reported the proteasomal degradation of Rad17 protein in the checkpoint recovery, the precise regulatory mechanisms of the degradation were not revealed because of the lack of identification of the destruction box. Our identification of the destruction boxes in Rad17 protein is expected to pave the way for further exploration of the regulatory mechanisms of the Rad17 degradation, which is involved in not only the checkpoint recovery but also the regulation of the basal activity of the ATR pathway.

## Experimental procedures

### Cell culture

COS-1 and HeLa cells were maintained in Dulbecco's modified Eagle medium (Nissui Pharmaceutical, 05919) supplemented with 5% bovine serum and 1% fetal bovine serum. COS-7 cells were maintained in Dulbecco's modified Eagle medium supplemented with 5% fetal bovine serum.

### Plasmids

The pcDNA3 vector encoding flag-tagged human Rad17 full-length protein (isoform 1, NCBI NP_579921.1) was described previously (14). Rad17 E295–D380 peptide was cloned into a C-terminus of EGFP in pcDNA4-TO to construct a plasmid vector expressing EGFP fused with Rad17 E295–D380 peptide. A flag-Rad17 M1–D82 peptide was cloned into pET29b in frame with an N-terminal S tag and a C-terminal histidine tag. pGEX-6P-Cdh1wt (570) was a gift from Jonathon Pines (Addgene plasmid # 39877; http://n2t.net/addgene:39877; RRID:Addgene_39877). The pcDNA3 vectors encoding the flag-EGFP-Rad17 full-length protein were constructed by inserting EGFP between flag tag and Rad17. The pcDNA3 vector encoding the flag-S35–G66-EGFP-E295–D380 protein was constructed as follows: The Rad17 E295–D380 peptide was fused at the C-terminus of flag-EGFP, and the Rad17 S35–G66 peptide that contains the tandem destruction boxes was inserted between the N-terminal flag tag and EGFP. The S35 − G66 sequence contains E46/S47 to Glycine and Q63/I64 to Leucine substitutions.

### Antibodies

The following antibodies were used: mouse anti-FLAG/M2, Sigma-Aldrich, F1804; rabbit anti-FLAG, Medical & Biological Laboratories, PM020; goat anti-FLAG, Novus Biologicals, NB600-344; Anti-GST, Medical & Biological Laboratories, PM013; anti-Hsc70, Santa Cruz Biotechnology, sc-7298; anti-NPT2, Abcam, ab33595; anti-Rad1, Santa Cruz Biotechnology, sc-14314/N-18; anti-Rad17, Sigma-Aldrich, R8654; and anti-Rad17-S645, Bethyl Laboratories, A300-153A.

### Fluorescence microscopy

Immunofluorescence staining was performed as described previously (14, 31). The localization of flag-Rad17 full-length protein was examined as follows. COS-1 cells were transfected with pcDNA3 plasmids encoding a flag-Rad17 full-length protein with the acidified polyethylenimine (32). Forty-eight hours after transfection, the cells were fixed with 4% paraformaldehyde. The cells were permeabilized and blocked with PBS (−)/0.1% saponin/3% BSA and stained with anti-FLAG/M2 antibody and goat anti-mouse antibody conjugated with Alexa Fluor 647 (Thermo Fisher Scientific, A21236) in an immuno-enhancer (Fujifilm Wako Pure Chemical Corporation). The cells were treated with 200 μg/ml RNase A for 1 h and stained with 5 μg/ml propidium iodide for 30 min. The data were obtained with confocal laser scanning microscopes Olympus Fluoview FV500 and Carl Zeiss LSM 700. The localization was classified into three groups: The flag-Rad17 protein was mostly localized in the nucleus (nucleus, Nuc), equally distributed in the nucleus and the cytoplasm (cytosol and nucleus, N/C), and mostly localized in the cytoplasm (cytosol, Cyto). The ratios of the three groups were calculated in each experiment, and the mean ± standard deviation was calculated and represented by the graphs. Error bars represent standard deviation. The *p*-value of more than 0.05 was noted as not significant (NS). The total number of cells and the number of cells classified in N, N/C, and C groups were summarized from more than two independent experiments, and the Chi-square values were calculated based on the total number of cells for indicated pairs of the flag-Rad17 constructs. The *p*-values were calculated based on the Chi-square distribution and the degrees of freedom.

COS-7 cells were transfected with 0.5 μg of pcDNA4/EGFP-Rad17 E295–D380 peptide using Lipofectamine 2000 (Thermo Fisher Scientific). Twenty-four hours after transfection, the cells were fixed with 2% paraformaldehyde. DNA was stained with 1 μM Hoechst 33342 in PBS (−)/3% BSA/0.1% saponin. Fluorescence microscopic images were captured with an IX83 inverted fluorescence microscope (Olympus).

HeLa cells were transfected with 1 μg of pcDNA3/flag-EGFP-Rad17 full-length protein using the acidified polyethylenimine. Forty hours after transfection, the cells were fixed with 2% paraformaldehyde. The cells were treated with 200 μg/ml RNase A for 1 h and stained with 5 μg/ml propidium iodide for 30 min. The data were obtained with Carl Zeiss LSM 700, and the EGFP signals were quantitated in the nucleus and the cytoplasm with Zen 3.2 (blue edition). The average intensity was used to calculate the cytoplasm/nucleus ratio of Rad17 protein amount, and means ± standard deviation was presented. In the box-and-whisker plots, the boxes indicate 25th, 50th, and 75th percentiles, and the whiskers indicate the smallest and largest data points excluding any outliers. The box-and-whisker plots and the dot plots were written with Seaborn ver. 0.11.1 and Matplotlib ver. 3.3.4 in Anaconda3 ver. 4.9.2.

### UV irradiation and Rad17-S645 phosphorylation

Rad17-S645 phosphorylation after UV irradiation was examined by immunoprecipitation followed by western blot as described previously (14).

### Stability of flag-Rad17 full-length protein

COS-1 cells were seeded in a 35 mm dish and transfected with 1 μg of pcDNA3 plasmids using the acidified polyethylenimine (32). The plasmids encode a flag-Rad17 full-length protein and neomycin phosphotransferase 2 (NPT2). The medium was replaced with fresh medium 6 to 10 h and 24 to 36 h after transfection. The cells were lysed with Laemmli SDS-PAGE sample buffer 48 to 54 h after transfection. Where noted, the cells were exposed to MG132, leptomycin B, or UV irradiation. The pcDNA4-TO plasmid, which lacks the NPT2 expression cassette, was used as the control vector. The lysate was probed with anti-FLAG and anti-NPT2 antibodies. The data were obtained with ChemiDoc XRS Plus (Bio-Rad), and the band intensity was quantitated with Quantity One (Bio-Rad). The intensity of anti-FLAG blot was normalized to that of anti-NPT2 blot, and the FLAG/NPT2 ratio was calculated. The graphs represent mean ± standard deviation, and error bars represent standard deviation. The lysate with flag-Rad17 wild type was prepared as a duplicate in each experiment, and the average intensity was used as 100% standard. The stability of the flag-S35–G66-EGFP-E295–D380 protein was examined in the same manner.

### Coprecipitation of Rad1 with flag-Rad17

Coprecipitation of Rad1 with flag-Rad17 was examined exactly as described previously (7, 14). The flag-Rad17 protein was precipitated from the low-salt extract, and coprecipitation of endogenous Rad1 was examined. The extract of flag-Rad17 wild type was prepared as a duplicate in each experiment. The coprecipitation efficiency of endogenous Rad1 was 0.5 to 1% (7, 14).

### Coprecipitation of GST-Cdh1 and flag-Rad17 M1–D82 peptide

Recombinant proteins of GST-Cdh1 and flag-Rad17 M1–D82 peptide were separately expressed in BL21(DE3), and bacterial lysates were prepared as described previously (9). The bacterial lysate containing GST-Cdh1, the lysate containing flag-Rad17 M1–D82, and M2 agarose (Sigma-Aldrich) were mixed and rotated for 12 to 17 h in a cold room. The resins were washed with buffer [20 mM NaPi (pH 6.8), 120 mM NaCl, 0.1% Triton X-100], and the coprecipitation of GST-Cdh1 was examined with anti-GST blot.

### Multiple alignment

The sequences of experimentally characterized destruction boxes were adopted from the APC/C degron repository (http://slim.ucd.ie/apc/index.php), where the references to known destruction boxes were also available. The destruction boxes used in the alignment were reported in the following references: Cyclin B1 (33), FoxM1 (34), MYF5 (35), Cdc6 (36), Hmmr (37), Plk1 (38), Cyclin A2 (39), Cyclin B3 (40), Fbxw5 (41), Securin (42), Geminin (43), and Aurora B (44).

### Structural modeling and molecular dynamic simulations

Human Rad17 was modeled based on X-ray crystallographic data of RFC1–PCNA complexes in *Saccharomyces cerevisiae* (PDB code: 1SXJ) (6). Human Rad17 N77–N338 was aligned with RFC1 D295–T530. Rad17 N339–D380 was not conserved in the RFC subunits and excluded from the modeling. We used a C-terminal domain of RFC1 (I531–G693) instead of that of Rad17, and Rad17 N77–N338 was fused with the C-terminal domain of RFC1. Rad17 L243 is in a sensor 1 sequence of AAA+ ATPase (11), and the sensor 1 sequence of Rad17 was aligned with that of RFC1. PCNA was also replaced with the 9-1-1 complex.

Structural modeling of human Rad17 was performed in a similar manner to the previous work (45). Briefly, the initial structure of Rad17 was built by a homology modeling software, Modeller ver. 9 (46). The complex model was placed in a periodic boundary box solvated with water molecules. Energy minimization was executed for the whole calculation system, and subsequently the temperature was elevated to 310 K under the constant volume condition. Then, molecular dynamics (MD) simulation was executed for 100 ns at the constant pressure of 1 atm and the constant temperature of 310 K. The PMEMD module of AMBER16 was used for the MD simulation (47). The AMBER force fields, FF14SB and GAFF2, were used for protein models and small organic molecules, respectively.

## Data availability

### Dataset

APC/C degron repository. http://slim.ucd.ie/apc/index.php [accessed 24 February 2020].

PhosphoSitePlus. https://www.phosphosite.org [accessed 24 February 2020].

## Supporting information

This article contains supporting information.

## Conflict of interest

The authors declare that they have no conflicts of interest with the contents of this article.

## Supporting information

Suppelemntal Figures S1–S5
