# Supplementary material for: Nuclear translocation promotes proteasomal degradation of human Rad17 protein through the N-terminal destruction boxes
Source: J Biol Chem. 2021 Jun 24;297(2):100831. doi: 10.1016/j.jbc.2021.100831 (PMC8318897; doi:10.1016/j.jbc.2021.100831)
Supplement: Suppelemntal Figures S1–S5 [file mmc1.pdf]

Supporting information

Nuclear translocation promotes proteasomal degradation of human Rad17 protein through N-terminal destruction box

Yasunori Fukumoto, Masayoshi Ikeuchi, Liang Qu, Tyuji Hoshino, Naoto Yamaguchi, Yuji Nakayama, Yasumitsu Ogra

Figure S1. Domain structure of human Rad17 protein.

Figure S2. The multiple sequence alignment of Rad17 proteins and RFC subunits reveals the central basic region of Rad17.

Figure S3. Subcellular localization of flag-EGFP-Rad17 full-length protein in HeLa cells.

Figure S4. The half-life of the Rad17 wild type and the K/R359–363A proteins.

Figure S5. The 230–270 deletion abolished the central part of the AAA+ ATPase domain of Rad17 protein.

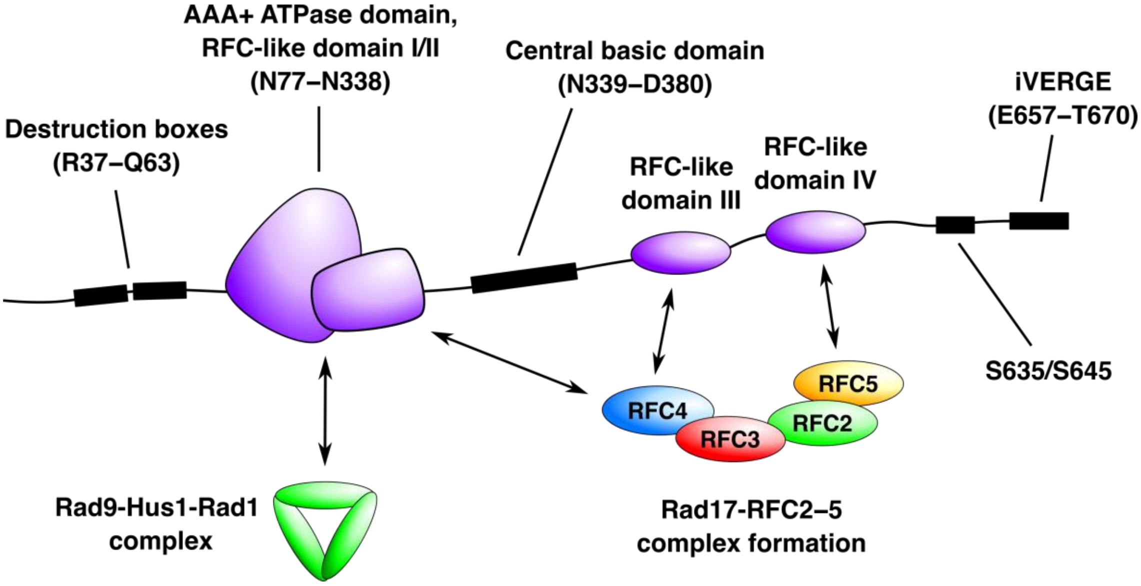

Figure S1. Domain structure of human Rad17 protein.

The domain structure of human Rad17 was mapped on Rad17 isoform 1 (NCBI NP_579921.1, 670 a.a.). RFC domains I, II, III, and IV were defined in the reference [6]. The domains and their corresponding references are as follows: R37–Q63, destruction boxes (this work); N77–N338, AAA+ ATPase domain (RFC-like domain I/II) [10, 11]; N339–D380, central basic domain (this work); D381 and later, multi α-helices (RFC-like domains III and IV) [6]; S635/S645, ATR phosphorylation sites [1]; and E657–T670, iVERGE (the polyanionic C-terminal tail) [7]. The ATPase domain interacts with the Rad9-Hus1-Rad1 complex, and RFC-like domains III and IV interact with the RFC subunits.

```
                                                              Central basic domain (N339–D380)
                         ATPase domain (N77–N338)
                                                                          K/R359–363
Hs. Rad17 308 KTSLELLCQG CSGDIRSAIN SLQFSSSKGE N--NL---RP RKKG-MSLKS D--AVLSKSK RRKKPDRVFE -NQEVQAIGG KDVSLFLFRA LGKIL---
Mm. Rad17 318 KTSLELLCQG CSGDIRSAIN SLQFSSSKGE N--SS---WS KKKR-MSLKS D--AAISKSK QKKKHNSTLE -NQEIQAIGG KDVSLFLFRA LGKIL---
Gg. Rad17 326 RASLELLCKG CSGDIRSAIN SLQFFSMKDC S--LEKDFWS RKKRSSTLKS ETAAAMCSLR KKSKSD-ISE -DQAIQAIGG KDASIFLFHA LGKII---
Xl. Rad17 313 KASLELICTG SSGDIRSAIN SLQFSARQGS S--LKNDSWS KSRG-KASKS GKP--SSKSK TKKEPRKGGE -NVDEVQAIG GKDASLFLFR ALGKIL--
Dr. Rad17 306 KAALDLLCSG SSGDIRSAIN SLQFSSFTDN S--LERRLWA SKKG-KSSSA -KP--AVKAK GRSKSSKSKD MQDESPAIGG KDASLFLFRA LGKIL---
Hs. RFC1  797 PPAMNEIILG ANQDIRQVLH NLSMWCARSK A--LTYDQAK ---------- ---------- ---------- ---ADSHRAK KDIKMGPFDV ARKVFAAG
Mm. RFC1  781 PPAMNEIILG ANQDVRQVLH NLSMWCAQSK A--LTYDQAK ---------- ---------- ---------- ---ADSQRAK KDIRLGPFDV TRKVFAAG
Xt. RFC1  787 PPAMNEIILG ANQDIRQVLH NLSMWCARSK A--LTYDEAK ---------- ---------- ---------- ---SSATNAK KDIKMGPFDV VRKVFSSG
Dr. RFC1  789 PPALNEVILA SNQDIRQVLH NLSMWSAKDK V--MTYDQAK ---------- ---------- ---------- ---ADANNAK KDMKLGPFDV CRKVFALG
Hs. RFC2  215 DDGLEAIIFT AQGDMRQALN NLQSTFSGFG F--INSEN-- ---------- ---------- ---------- ---------- ------VF-- --------
Mm. RFC2  133 DDGLEAIIFT AQGDMRQALN NLQSTFSGFG Y--INSEN-- ---------- ---------- ---------- ---------- ------VF-- --------
Gg. RFC2  220 DDGLEAIIFT AQGDMRQALN NLQSTYSGFG F--INSEN-- ---------- ---------- ---------- ---------- ------VF-- --------
Dr. RFC2  210 NDGLEAIIFT AQGDMRQALN NLQSTNSGFG Y--INSEN-- ---------- ---------- ---------- ---------- ------VF-- --------
Hs. RFC3  209 SQLAHRLAEK SCRNLRKALL MCEACRVQQY P--FTADQEI ---------- ---------- ---------- ---------- ---------- --------
Mm. RFC3  209 STLARRLAEK SCRNLRKALL MCEACRVQQY P--FTEDQEI ---------- ---------- ---------- ---------- ---------- --------
Xt. RFC3  209 QELARKIAEK SGRNLRKALL MCEACRVQQY P--FSADQDL ---------- ---------- ---------- ---------- ---------- --------
Dr. RFC3  209 PELAKQIAEK SGRNLRKALL MCEACRVQQY P--FSPDQDI ---------- ---------- ---------- ---------- ---------- --------
Hs. RFC4  224 DEGIAYLVKV SEGDLRKAIT FLQSATRLTG GKEITEKV-- ---------- ---------- ---------- ---------- ------IT-- --------
Mm. RFC4  224 NEEIAYLVKI SEGDLRKAIT FLQSATRLTG GKEVSEDV-- ---------- ---------- ---------- ---------- ------IT-- --------
Xt. RFC4  223 NEAISCLVEV SEGDLRKAIT FLQSATRLTG GKEITEEI-- ---------- ---------- ---------- ---------- ------VT-- --------
Dr. RFC4  219 TEGVDALVRV SEGDLRKAIT FLQSGARLNS EREITEQT-- ---------- ---------- ---------- ---------- ------II-- --------
Hs. RFC5  199 EDGMKALVTL SSGDMRRALN ILQSTNMAFG -K-VTEET-- ---------- ---------- ---------- ---------- ------VY-- --------
Mm. RFC5  198 EDGMKALVTL SSGDMRRALN ILQSTNMAFG -K-VTEET-- ---------- ---------- ---------- ---------- ------VY-- --------
Xl. RFC5  194 PDGMKALVTL SNGDMRRSLN ILQSTNMAYG -K-VTEDT-- ---------- ---------- ---------- ---------- ------VY-- --------
Dr. RFC5  193 PDGMKAIVTL STGDMRRSLN ILQSTHMAYG -K-VTEET-- ---------- ---------- ---------- ---------- ------VY-- --------
```

Figure S2. Multiple sequence alignment of Rad17 proteins and RFC subunits reveals the central basic region of Rad17.

The alignment shows the C-terminal regions of ATPase domains in vertebrate Rad17 and RFC proteins. Human Rad17 N77–N338 residues encode the ATPase domain, and Rad17 D380 and later residues encode the C-terminal α-helical region. The central basic region resides between N339 and D380, and the bracket indicates the Rad17 K/R359–363 residues, which encode a part of the putative nuclear localization signal.

Hs, *Homo sapiens*. Mm, *Mus musculus*. Gg, *Gallus gallus*. Xl, *Xenopus laevis*. Xt, *Xenopus tropicalis*. Dr, *Danio rerio*.

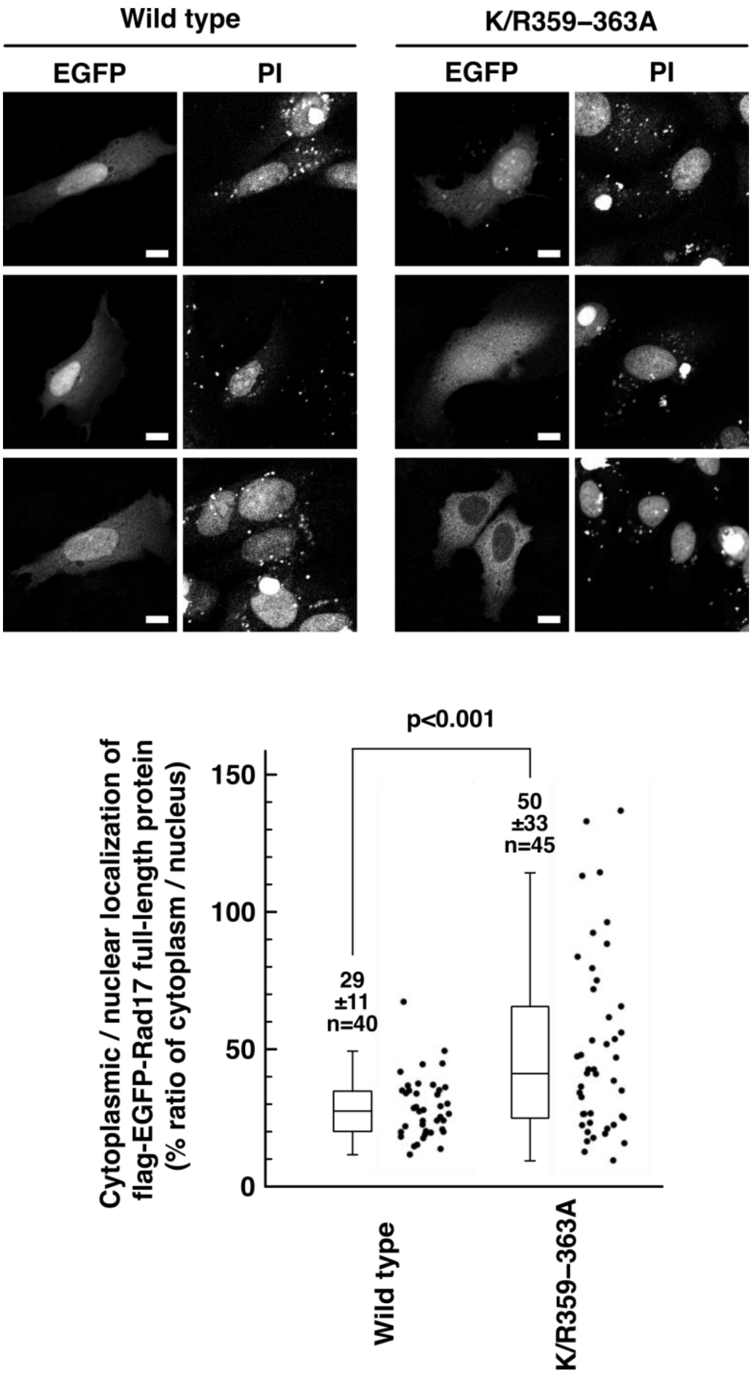

Figure S3. Subcellular localization of flag-EGFP-Rad17 full-length protein in HeLa cells.

HeLa cells were transfected with pcDNA3 vectors expressing the flag-EGFP-Rad17 full-length protein. Forty-eight hours after transfection, the cells were fixed and stained with propidium iodide. Scale bars: 10 μm. The intensities of EGFP signals in the nucleus and the cytoplasm were quantitated, and the cytoplasmic/nuclear localization ratios were calculated. The graph shows results from two independent experiments. The p-value was calculated using Welch's *t*-test. n indicates the number of cells.

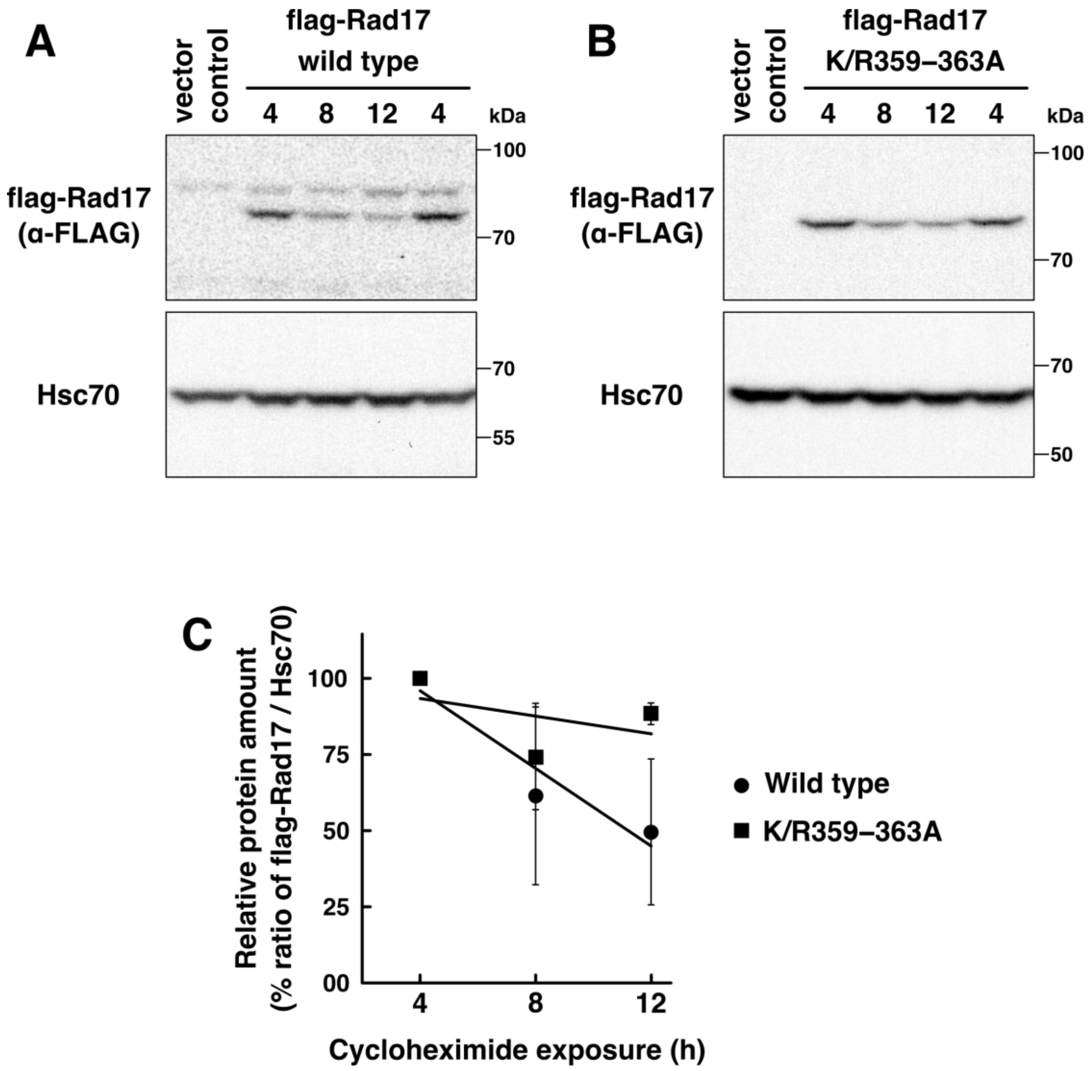

Figure S4. The half-life of the Rad17 wild type and the K/R359–363A proteins.

The Rad17 wild type (A) and the K/R359–363A (B) proteins showed a half-life of 7.9 and 34.7 hours. COS-1 cells were transfected with plasmid vectors expressing the flag-Rad17 protein. At 28–32 hours after transfection, the cells were split into three dishes. From 44–48 hours after transfection, the cells were exposed to 300 μg/mL cycloheximide for the indicated period. The SDS-lysate was prepared and probed with the indicated antibodies. The signal intensity of anti-FLAG blot was normalized to anti-Hsc70 blot. For the 4 hours exposure, the same sample was applied to both sides and used as 100% control. The graph represents results from three independent experiments (C). The half-life was calculated as the slope of the linear regression.

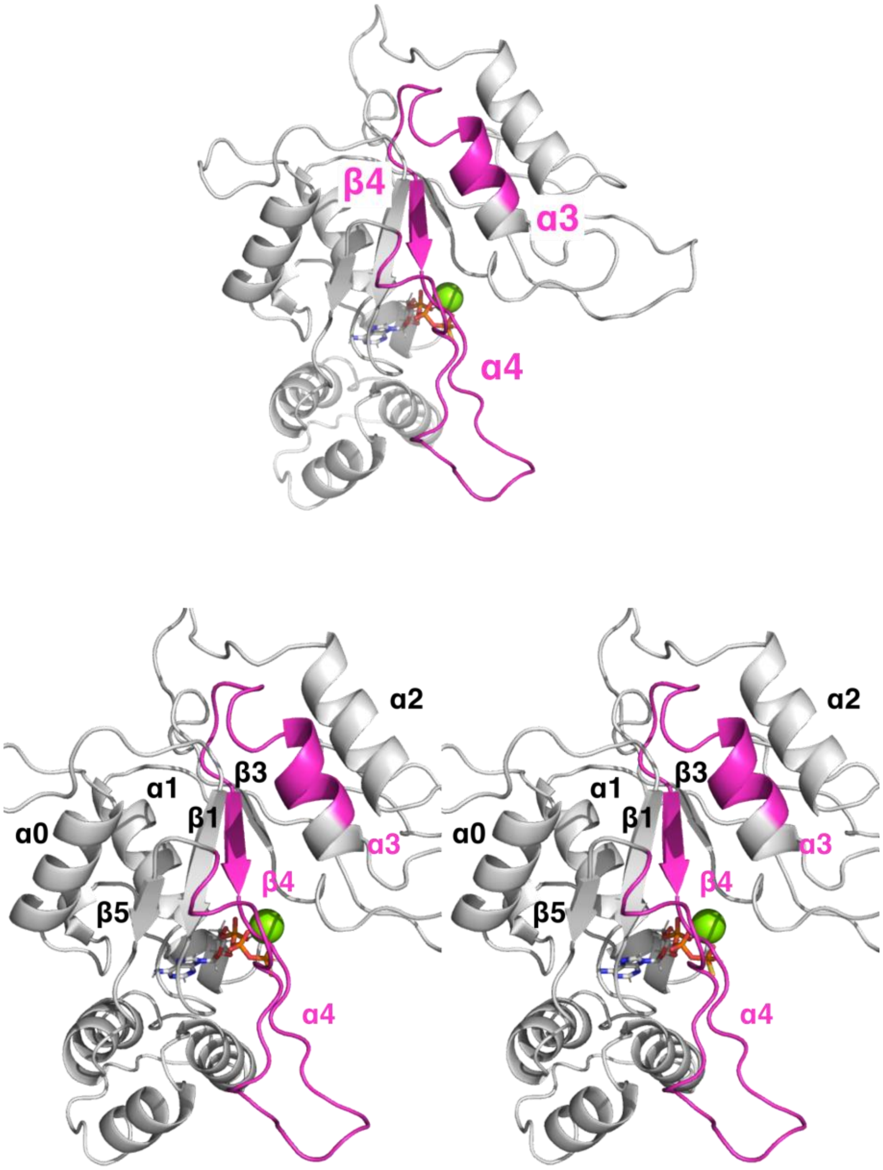

Figure S5. The 230–270 deletion abolished the central part of the AAA+ ATPase domain of Rad17 protein.

The Rad17 230–270 residues were mapped in the ATPase domain of human Rad17 (N77–N338) on a structural model that was built on the basis of the homology with RFC1. The 230–270 residues are shown in red. The Δ230–270 abolished the α3-helix, the β4-strands, and the α4-helix of the canonical AAA+ ATPase domain. The β4-strand is located at the center of the central β-sheet that is composed of β1–β5 strands. The β2-strand is located behind the β3-strand. The α-helices and the β-strands were annotated according to the reference [28].
